# Evaluation of the VA Medical Foster Home Program: Factors Important for Expansion and Sustainability During COVID19

**DOI:** 10.1093/geroni/igaa057.3456

**Published:** 2020-12-16

**Authors:** Nelly Solorzano, Chelsea Manheim, Leah Haverhals, Cari Levy

**Affiliations:** 1 Denver Research Institute, Aurora, Colorado, United States; 2 Rocky Mountain Regional Medical Center, Aurora, Colorado, United States; 3 Department of Veterans Affairs, Denver, Colorado, United States; 4 VA Eastern Colorado Health Care System, Aurora, Colorado, United States

## Abstract

In 2020, the Center of Innovation for Veteran Centered and Value Driven Care (COIN) continued its monitoring and evaluation of the Veterans Administration (VA) Medical Foster Home (MFH) programs expansion into rural areas. Veterans in MFHs are provided 24/7 care by VA trained and supervised community caregivers and primary care by VA Home Based Primary Care (HBPC) teams. One year after the three-year (2017-2019) expansion funds stopped, COIN continued monitoring remaining programs. Objectives were to understand factors critical for program expansion and sustainability and the impact of COVID-19. Phone interviews were conducted with sixteen coordinators from seventeen programs. A thematic analysis approach was used to address the evaluation objectives using transcript data. Findings showed factors important to program sustainability were: 1) Program fit (finding caregivers in the community); and 2) Local VA facility support (staffing, adaptation, and local leadership support). COVID prompted losing some caregivers and prevented others from joining. Program staffing was not impacted as many program activities ceased. Recreational therapists (RTs) were significant to maintaining Veterans well-being and reducing social isolation through virtual activities. COVID required coordinators transition their supervision of MFHs to new virtual environments and HBPC to increase telehealth to new levels. Local leadership became important to monitoring local conditions and providing support to programs. The evaluation: 1) Found factors important to program sustainability were also critical to keeping programs operational during the pandemic; and 2) Stimulated future research on the suitability of MFH programs to meet challenges to resurgences of COVID or other national emergencies.

